# ECM-Syndecan-4-FAK signaling is associated with epithelial invagination and buccolingual asymmetry during mandibular molar development

**DOI:** 10.3389/fphys.2026.1805860

**Published:** 2026-05-19

**Authors:** Yuanjing Jiang, Chuanqing Mao, Rui Kong, Mingying Hu, Jiaqi Zhou, Chengyan Ren, Weihui Chen

**Affiliations:** 1 Department of Oral and Maxillofacial Surgery, Fujian Medical University Union Hospital, Fuzhou, Fujian, China; 2 Hospital and School of Stomatology, Fujian Medical University, Fuzhou, Fujian, China; 3 Stomatological Hospital of Xiamen Medical College, Xiamen, Fujian, China

**Keywords:** epithelial invagination, extracellular matrix-basement membrane remodeling, focal adhesion kinase (FAK)/pFAK signaling, single-cell RNA sequencing (scRNA-seq), syndecan-4 (Sdc4), tooth morphogenesis

## Abstract

**Introduction:**

Tooth morphogenesis in mice provides valuable insights into the cellular and molecular mechanisms governing organ shape. During the critical transition from the dental placode to the early cap stage (E12.5–E14.5), epithelial invagination and shape changes are accompanied by complex cellular rearrangements, including suprabasal intercalation and collective cell behaviors. This study examined how Syndecan-4 (Sdc4), focal adhesion kinase (FAK), and basement membrane-related interactions are deployed during early mandibular first molar development.

**Methods:**

We performed a stage-matched reanalysis of publicly available single-cell RNA-seq datasets, together with morphometric and spatial validation, to characterize epithelial state changes across E12.5–E14.5.

**Results:**

At E12.5, DE-like epithelial cells displayed a proliferative odontogenic program enriched for cell-cycle regulators and key signaling pathways, including Wnt and Hippo. By E13.5, extracellular matrix components, including collagen, laminin, and agrin, together with focal adhesion-related pathways, became prominent in association with epithelial remodeling. At E14.5, upregulation of adhesion and structural scaffold components was consistent with tissue stabilization during morphogenesis. Across these stages, the data suggest a temporal shift from proliferative odontogenic activity to ECM-adhesion remodeling and then to tissue stabilization during early molar morphogenesis.

**Discussion:**

Integrating the transcriptomic, morphometric, and spatial findings, we propose that an ECM-Sdc4-FAK-related signaling axis may contribute to epithelial invagination and the emergence of buccolingual asymmetry. More broadly, these findings highlight cell-matrix interactions as an important component of the early tooth morphogenetic environment, while functional perturbation studies will be needed to establish direct causality.

## Introduction

Tooth morphogenesis provides a model for understanding how signaling, cell behaviors, and extracellular matrix (ECM) constraints contribute to organ shape. In the mouse mandibular first molar (M1), the odontogenic epithelium progresses rapidly from dental placode and bud morphogenesis (E12.5-E13.5) to early cap morphology (E14.5), coinciding with epithelial stratification, epithelial-mesenchymal coupling, and the emergence of signaling and structural compartments that precede cusp formation (Lesot et al., 2014; Yu and Klein, 2020). Recent studies suggest that epithelial invagination during this period is not solely driven by growth. Analyses of the bud-to-cap transition show that this process is largely independent of proliferation. Inhibition of focal adhesion kinase (FAK) arrests molar explants at the bud stage, suggesting basal cell-matrix coupling as critical for shape change (Yamada et al., 2019). Thus, matrix engagement plays a key role in epithelial behavior during invagination. Studies highlight that ectodermal organ invagination can also be driven by suprabasal cell intercalation and contractile behaviors, emphasizing collective cell rearrangements as morphogenetic drivers (Mogollón et al., 2021). Experimental uncoupling of epithelial stratification and placode invagination further indicates that early tooth shape is controlled by distinct cellular programs, not by a single growth-based mechanism (Li et al., 2016). Live imaging revealed “vertical telescoping” as a strategy for invagination, where vertical cell movements across the epithelial sheet generate bending without apical constriction as the sole driver (Li et al., 2020).

These morphogenetic behaviors are linked to developmental signals via cytoskeletal and adhesion effectors. Shh signaling controls non-muscle myosin II activation, promoting suprabasal movements and cell-cell adhesion, connecting a patterning pathway to force-generating and adhesion-regulating mechanisms during invagination (Du et al., 2024). Intraepithelial migration also contributes to the proper positioning and initiation of the molar primordium, further emphasizing cell movement in odontogenesis (Prochazka et al., 2015). Together, these studies suggest that tooth invagination arises from coordinated cell rearrangements and force transmission across epithelial layers. The basement membrane and its receptors coordinate cellular programs with tissue-scale geometry. A landmark study demonstrated that laminin α5 is essential for dental epithelial growth and polarity, implicating laminin-integrin coupling in determining tooth germ size and shape (Fukumoto et al., 2006). Syndecans are spatiotemporally regulated during odontogenesis, consistent with roles in epithelial-mesenchymal coupling and ECM remodeling. In murine tooth germs, syndecan expression is stage- and compartment-specific from bud to bell stages (Wu et al., 2020), while in humans coordinated changes in syndecans and heparan-sulfate (HS) biosynthetic/degradative enzymes imply developmental tuning of HS-dependent ligand retention and presentation within the ECM niche. Among these, syndecan-4(Sdc4) is linked to dental epithelial differentiation and, as an HS proteoglycan co-receptor, may integrate ECM/growth-factor signals with cell-matrix adhesion to guide epithelial curvature, invagination, and rearrangements (Yan et al., 2014). In parallel, FAK couples mechanical inputs to cytoskeletal remodeling by regulating basement membrane remodeling and collagen I dynamics during invagination (Yamada et al., 2019; Le Coq et al., 2022).

However, how basement membrane composition, Sdc4 and focal adhesion/FAK activity are deployed across specific epithelial and mesenchymal cell states during the narrow E12.5-E14.5 window remains poorly resolved. Recent stage-resolved single-cell and spatially anchored resources for mouse molar development now enable time-resolved analyses of epithelial and mesenchymal states *in vivo* (Hu et al., 2022; Wang et al., 2022b). In the present study, we reanalyzed stage-matched public datasets with a focused emphasis on the epithelial compartment during the placode-to-cap transition. Our aim was to define how odontogenic epithelial states change over time, how epithelial-mesenchymal signaling is reorganized during invagination, and how these transcriptomic changes relate to spatial ECM remodeling and the emergence of buccolingual asymmetry. Rather than generating another descriptive atlas, we used these datasets to identify a basement membrane-Sdc4-FAK-related signaling framework during early mandibular molar morphogenesis.

## Materials and methods

### Animals and embryo collection

C57BL/6 mice were housed under specific pathogen-free (SPF) conditions at Fujian Medical University. Timed pregnancies were generated by pairing adult females (8–12 weeks old) with males overnight at a 2:1 ratio, and the morning a vaginal plug was detected was designated as embryonic day 0.5 (E0.5). Embryos were collected at E12.5, E13.5, and E14.5 for subsequent analyses. All animal procedures were performed in accordance with institutional guidelines and were approved by the Animal Ethics Committee of Fujian Medical University (IACUC FJMU, 2024-0228).

### Data acquisition and preprocessing

Publicly available single-cell RNA-seq (scRNA-seq) datasets of mouse craniofacial tissues at E12.5-E14.5 were retrieved from FaceBase (E12.5: Record ID 1-DTK2, DOI: 10.25550/1-DTK2; E13.5-E14.5: Record ID A-QVNG, DOI: 10.25550/A-QVNG). Raw count matrices and associated metadata were processed using Seurat (v4.1.1) in a uniform workflow. Low-quality cells and potential doublets were excluded by retaining cells with ≥200 detected genes and ≤15% mitochondrial transcripts. Dimensionality reduction was performed using principal component analysis (PCA), and cells were visualized with uniform manifold approximation and projection (UMAP).

### Study design and analytical scope

This study was designed as a hypothesis-driven reanalysis of publicly available, stage-matched scRNA-seq resources. We specifically focused on the epithelial compartment during the E12.5-E14.5 placode-to-cap transition. All datasets were processed using a unified workflow to enable stage-consistent comparisons of DE-like versus Non-DE-like epithelial states, epithelial-mesenchymal communication, and temporal shifts from proliferative to adhesion- and ECM-associated programs.

### Single-cell annotation and epithelial subclustering

**Figure 1:**
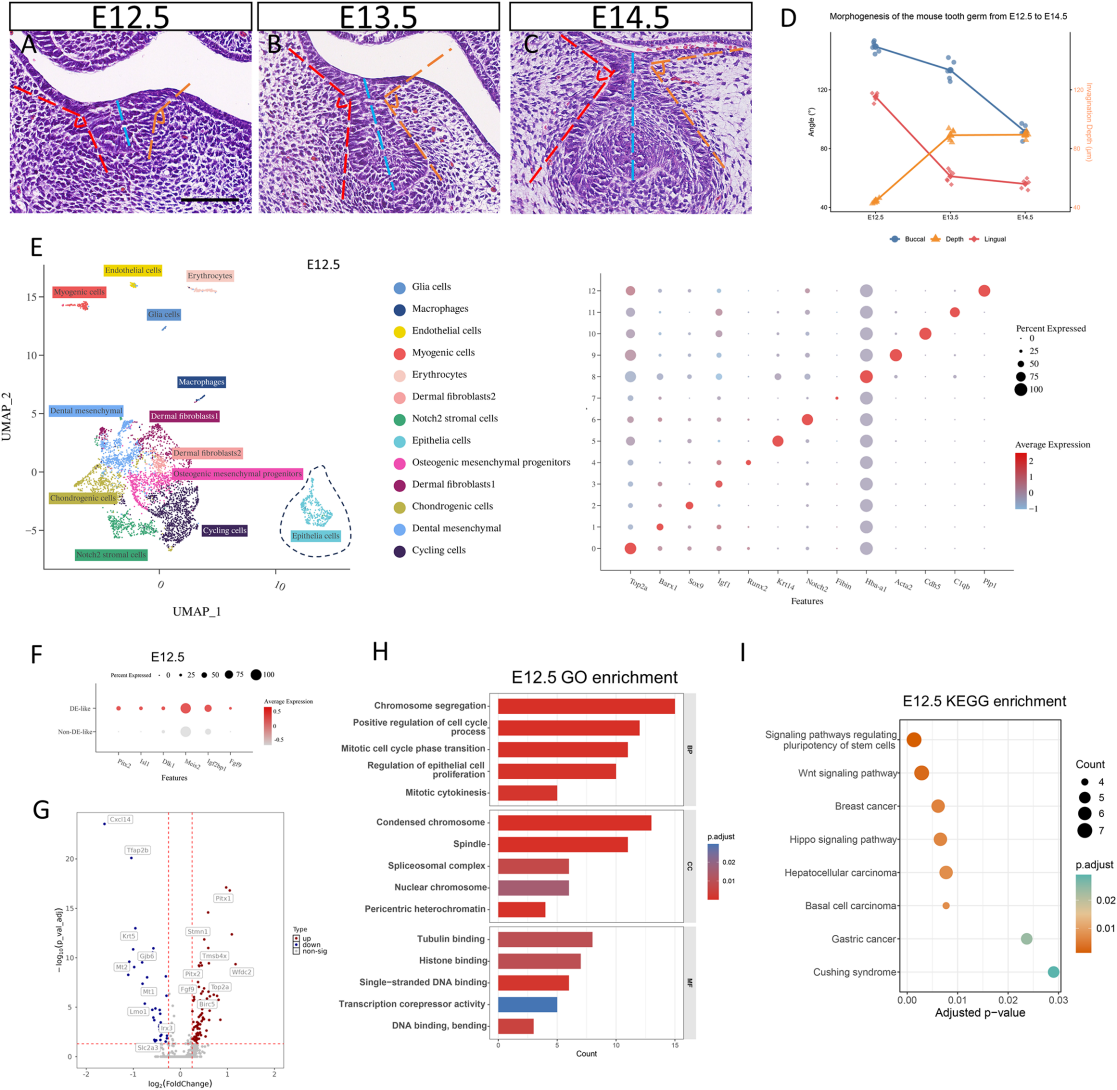
Early mandibular molar morphogenesis and transcriptomic characterization of E12.5 DE-like epithelium. **(A–C)** H&E staining shows the morphology of the mandibular first molar germ from E12.5 to E14.5. The blue dashed line indicates the invagination depth of the dental lamina/tooth germ complex. Orange and red angles denote the lingual and buccal cervical angles formed between the cervical region of the tooth germ and the adjacent oral epithelium, respectively. **(D)** Quantitative analysis of tooth germ morphogenesis from E12.5 to E14.5. **(E)** Cell types within the E12.5 mandibular primordium were annotated on the UMAP plot based on established differentiation marker genes. **(F)** Dot plot showing the expression of signature genes in the E12.5 DE-like cells. **(G)** Volcano plot of differentially expressed genes (DEGs) between the E12.5 DE-like and Non-DE-like cells. **(H, I)** GO and KEGG enrichment analyses of these DEGs.

**Figure 2:**
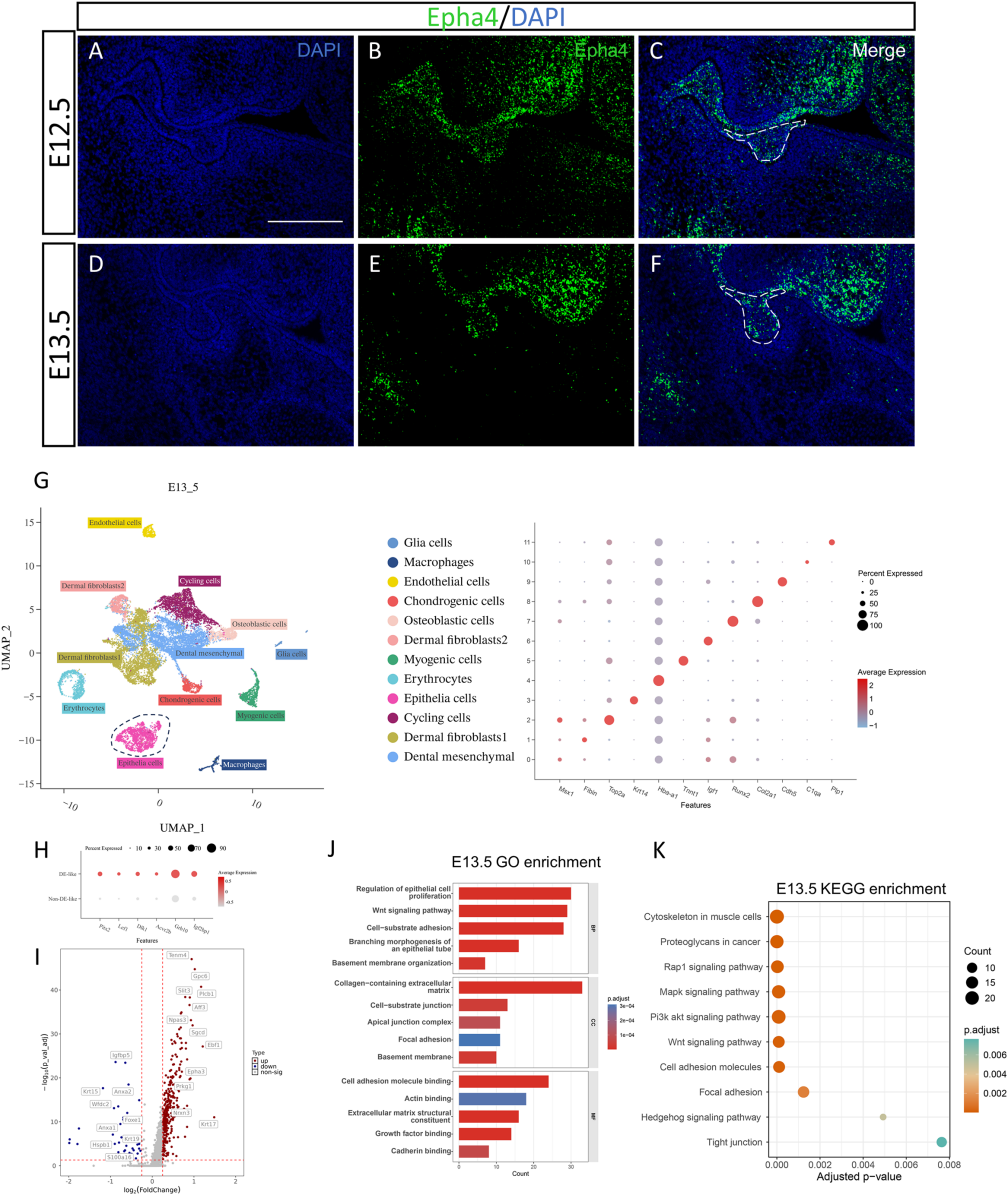
scRNA-seq reanalysis of the E13.5 mouse molar region. **(A-F)** *In situ* hybridization analysis of Epha4 mRNA expression in mouse molars at E12.5-E13.5. Dashed lines indicate the tooth germ region. Scale bar = 50μm. **(G)** Cell types in the E13.5 molar and surrounding tissues were annotated on the UMAP plot based on established differentiation marker genes. **(H)** Dot plot showing the expression of characteristic genes in DE-like cells at E13.5. **(I-K)** Volcano plots of differentially expressed genes between DE-like and Non-DE-like cells at E13.5, along with GO and KEGG enrichment analyses.

**Figure 3:**
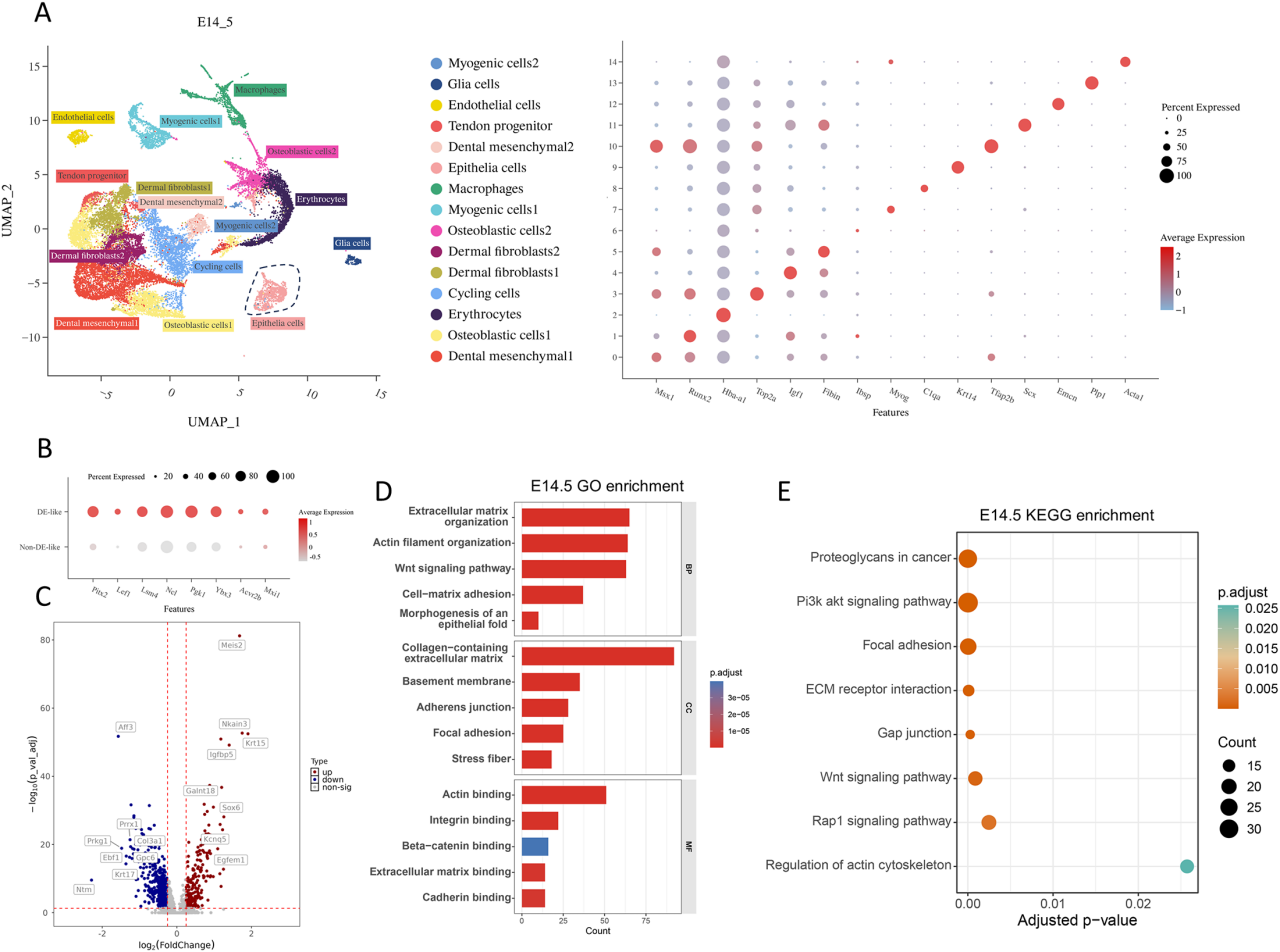
Differential gene expression analysis and pathway enrichment in DE-like and Non-DE-like cells at E14.5. **(A)** Cell types within the E14.5 molar and surrounding tissues were annotated on the UMAP plot based on established differentiation marker genes. **(B)** Dot plot showing the expression of characteristic marker genes in DE-like cells at E14.5. **(C-E)** Volcano plots of differentially expressed genes between DE-like and Non-DE-like cells at E14.5, together with GO and KEGG pathway enrichment analyses.

Epithelial cells were identified using canonical epithelial markers and subset for downstream analyses. Unsupervised clustering was performed within the epithelial compartment to resolve intralineage heterogeneity. Clusters were annotated as dental epithelium-like (DE-like) or Non-dental epithelium-like (Non-DE-like) based on published lineage-specific markers (Yuan et al., 2020; Hu et al., 2022; Wang et al., 2022b). DE-like and Non-DE-like populations showed largely mutually exclusive expression of signature genes, supporting the robustness of the annotation (**Figures 1F**, **2H**, **3B**).

### Differential expression analysis

Differentially expressed genes (DEGs) between the DE-like and Non-DE-like groups were identified using the Wilcoxon rank-sum test as implemented in Seurat (min.pct = 0.10, logfc.threshold = 0.25, adjusted P < 0.05). To ensure symmetric detection of DEGs, reciprocal comparisons were performed (DE-like vs. Non-DE-like).

### Functional enrichment analysis

Gene Ontology (GO) and Kyoto Encyclopedia of Genes and Genomes (KEGG) pathway enrichment analyses were performed using clusterProfiler (v4.2) with the org.Mm.eg.db annotation database. Terms were considered significantly enriched if they had an adjusted P value < 0.05 and contained at least five genes (maxGSSize = 500).

### Cell-cell communication analysis

Cell-cell communication among DE-like, Non-DE-like, and dental mesenchyme (DM) populations was analyzed using the CellChat package (v1.1.3). Highly variable ligand–receptor pairs were identified based on expression criteria, requiring expression in ≥10% of cells in at least one population. Communication networks were constructed and statistically evaluated using permutation-based significance testing. Signaling pathways with significantly increased interaction probabilities (FDR < 0.05) were visualized to delineate dominant signaling patterns across developmental stages. Trajectories of key developmental signaling families (BMP, FGF, WNT, SHH, and NOTCH) were systematically examined using network centrality metrics.

### ECM-remodeling protease and basement membrane gene survey

To investigate candidate mediators of local basement membrane remodeling, we surveyed the expression of ECM-remodeling proteases and basement membrane-associated genes across epithelial and mesenchymal compartments at E12.5-E14.5. Candidate genes were examined directly in the stage-matched scRNA-seq datasets on the basis of normalized expression patterns across major epithelial and mesenchymal cell populations, with particular attention to proteases previously implicated in ECM turnover and basement membrane remodeling.

### Histology

Embryos were fixed in 4% paraformaldehyde (PFA) and subsequently paraffin-embedded. Serial sections were prepared and stained with hematoxylin and eosin (H&E) according to standard protocols.

Immunohistochemistry and immunofluorescence

For immunohistochemistry, paraffin sections underwent antigen retrieval and were incubated with 3% hydrogen peroxide for 10 min to quench endogenous peroxidase activity. Sections were then incubated with an anti-Ki67 primary antibody (1:200; Abcam). A peroxidase-conjugated streptavidin goat anti-rabbit IgG secondary antibody (Abcam) was applied, and signals were developed using 3,3′-diaminobenzidine (DAB). Sections were counterstained with hematoxylin.

For immunofluorescence, paraffin sections were incubated with the following primary antibodies: collagen I (1:500; Abcam), collagen IV (1:500; Abcam), syndecan-4 (1:200; OriGene), and phospho-FAK (pFAK, 1:200; Invitrogen). Alexa Fluor–conjugated secondary antibodies were applied as appropriate. Nuclei were counterstained with 4′,6-diamidino-2-phenylindole (DAPI), and images were acquired using a fluorescence microscope(Olympus FV3000 confocal laser scanning microscope, Olympus, Japan).

### RNA fluorescence *in situ* hybridization

RNA fluorescence *in situ* hybridization (RNA-FISH) was performed on formalin-fixed, paraffin-embedded (FFPE) tissue sections using a commercial single-plex fluorescent RNA *in situ* hybridization kit (PinpoRNA™ 2.0, PIF1000; Pinpoease, China) according to the manufacturer’s instructions. Briefly, sections underwent RNA target retrieval, followed by hybridization with a gene-specific probe (Epha4) and signal amplification. Fluorescent signals were visualized by fluorescence microscopy(Olympus FV3000 confocal laser scanning microscope, Olympus, Japan), and nuclei were counterstained with DAPI.

### Statistical analysis

Data were obtained from six independent biological samples (n = 6) and are presented as the mean ± standard deviation (SD). Comparisons between two groups were performed using an unpaired Student’s t-test, whereas differences among multiple groups were assessed using one-way analysis of variance (ANOVA). A P value < 0.05 was considered statistically significant. The average optical density (AOD) of collagen I and the positive cell rate= (Number of positive cells/Total number of cells in a single field) * 100% were quantified using ImageJ software (v1.54g).

## Results

### Stage-matched scRNA-seq links morphogenesis to cellular programs

Using haematoxylin and eosin (H&E) staining of mouse mandibular first molar (M1) sections collected from E12.5 to E14.5, we observed a morphological progression from the dental lamina stage to the bud stage and subsequently to the early cap stage. To quantitatively assess epithelial remodeling during this period, we measured the invagination depth of the dental lamina/tooth germ complex and quantified the lingual and buccal cervical angles formed between the cervical region of the tooth germ and the adjacent oral epithelium (**Figures 1A–C**). From E12.5 to E13.5, the increase in invagination depth and the change in the lingual cervical angle were greater than those observed from E13.5 to E14.5. In contrast, the buccal cervical angle changed to a similar extent across the E12.5-E13.5 and E13.5-E14.5 intervals, while spanning a wide dynamic range across developmental stages. Collectively, these measurements suggest that early morphogenesis (E12.5-E13.5) is characterized by pronounced epithelial invagination and cervical narrowing, whereas later development (E13.5-E14.5) features attenuated invagination and the onset of bucco-lingual asymmetry, as evidenced by divergent trajectories of the lingual and buccal cervical angles (**Figure 1D**). To link these morphometric changes to the underlying cellular and molecular programs, we performed a uniform, stage-matched reanalysis of publicly available single-cell transcriptomic datasets from E12.5, E13.5, and E14.5. This analysis was specifically designed to enable direct cross-stage comparison of odontogenic epithelial states, rather than broad atlas-level description, and to identify candidate transcriptional and signaling modules associated with epithelial invagination and the onset of buccolingual asymmetry.

We reanalyzed publicly available single-cell RNA-seq datasets from E12.5, E13.5, and E14.5. After quality control and unsupervised clustering, we identified 13 clusters at E12.5 (**Figure 1E**), 12 clusters at E13.5 (**Figure 2G**), and 15 clusters at E14.5 (**Figure 3A**). Using established lineage markers and cluster-enriched genes (Yuan et al., 2020; Hu et al., 2022; Wang et al., 2022b; Ye et al., 2022), we annotated these clusters into 15 major cell types. Representative markers included cycling cells (*Top2a, Birc5, Ccnb2*), mesenchymal cells (*Msx1, Barx1, Tfap2b*), chondrogenic cells (*Sox9, Col2a1*), dermal fibroblasts (*Igf1, Fibin*), tendon progenitors (*Scx, Shox2*), myogenic cells (*Myog, Tnnt1*), endothelial cells (*Cdh5, Pecam1*), epithelial cells (*Krt14, Krt15, Epcam*), glial cells (*Plp1, Sox10*), macrophages (*C1qa, C1qb*), erythrocytes (*Hba-a1, Hbb-bs*), osteoblastic cells (*Runx2, Sp7, Alpl*), and Notch2+ stromal cells (*Notch2*). To investigate odontogenic epithelial invagination, we extracted the epithelial compartment for downstream analyses. Epithelial cells were classified into DE-like and Non-DE-like populations based on lineage-specific markers (**Figures 1F**, **2H**, **3B**). This stratification enabled differential expression analyses between invaginating DE-like cells and the adjacent Non-DE-like oral epithelium and provided a basis for subsequent stage-resolved cell-cell communication analyses.

### E12.5 DE-like epithelium exhibits a proliferative odontogenic program and prominent mesenchyme-to-epithelium signaling

At E12.5, comparison of DE-like and non-DE-like epithelial cells identified 123 differentially expressed genes (DEGs; FDR-adjusted P < 0.05), including 87 upregulated and 36 downregulated genes in DE-like cells (**Figure 1G**). DE-like cells displayed an enriched proliferative program, with higher expression of cell-cycle and mitotic regulators (e.g., *Top2a, Ube2c, and Birc5*), indicating elevated proliferative activity. DE-like cells also expressed higher levels of odontogenic and developmental regulators (e.g., *Pitx1/2, Sox2, and Dapl1*), consistent with an epithelial state primed for morphogenesis. In contrast, genes downregulated in DE-like cells included markers of general epithelial identity (e.g., *Krt5 and Tfap2b*) and gap junction components (*Gjb2 and Gjb6*), suggesting attenuation of a basal epithelial program (**Supplementary Table 1**). GO enrichment of the DEGs indicated overrepresentation of mitotic progression-related processes, including chromosome segregation, cell-cycle regulation, and cytokinesis. GO cellular component terms were enriched for mitotic structures, including spindle-related components and condensed chromosomes, consistent with a high fraction of cycling DE-like cells. Spliceosome-related terms were also enriched, suggesting increased RNA processing demands accompanying rapid proliferation (**Figure 1H**). KEGG pathway analysis further highlighted signaling programs implicated in epithelial growth and developmental regulation, including pathways regulating pluripotency, Wnt signaling, and Hippo signaling. Disease-associated categories (e.g., basal cell carcinoma) were also enriched, likely reflecting shared proliferation-associated transcriptional programs (**Figure 1I**).

**Figure 4:**
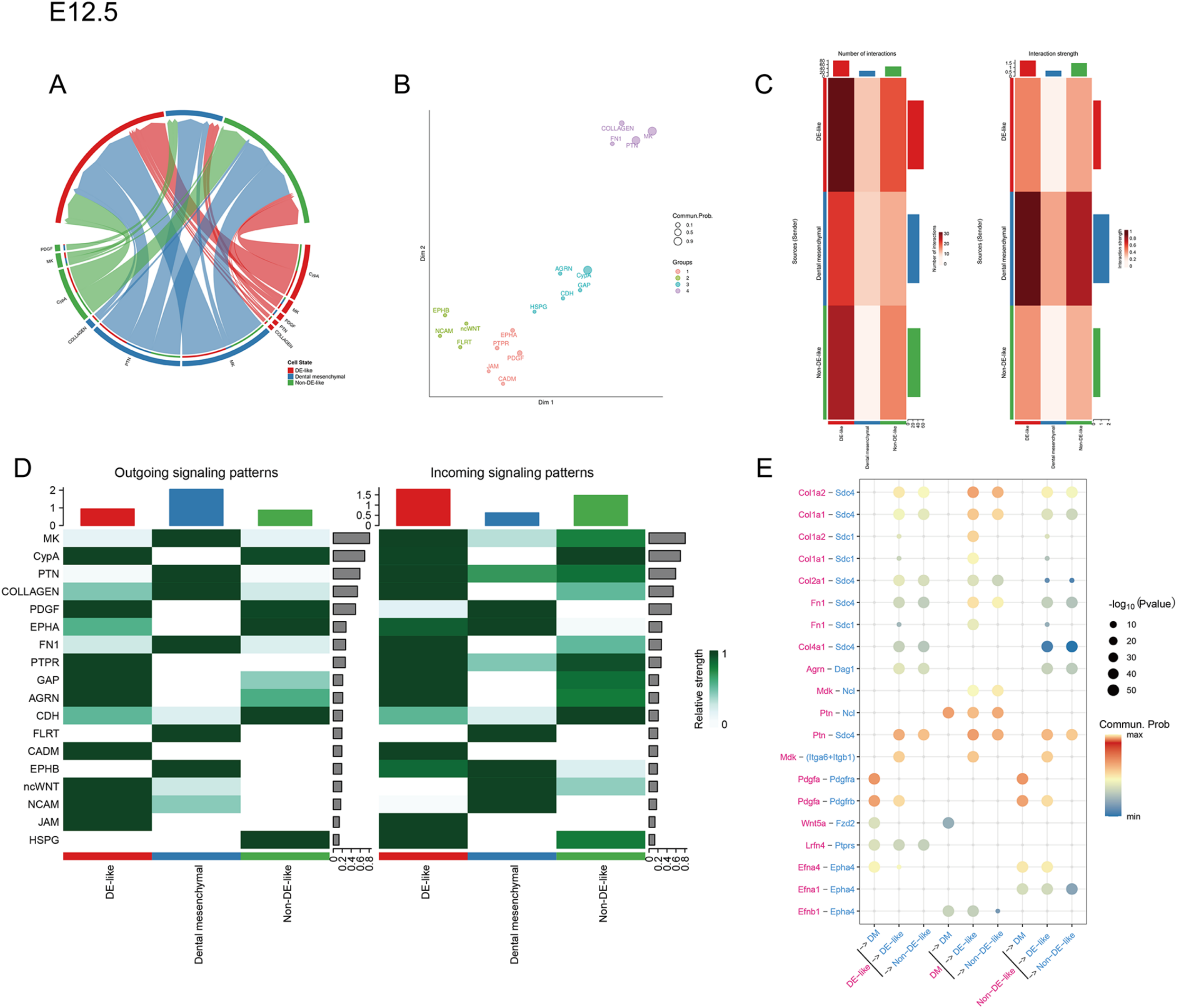
ECM-associated intercellular communication and ligand-receptor networks at E12.5. **(A)** Chord diagram showing signaling pathways with significant intercellular communication probabilities (p < 0.05) from sender to receiver cell groups. Each sector of the outer ring represents a pathway; wider chords indicate higher communication probabilities. Colors denote cell groups, and arrows point from signaling sources to targets. **(B)** Each dot represents a signaling pathway; shorter distances indicate greater similarity in their communication patterns. **(C)** Heatmap of inferred intercellular communication networks among major cell compartments at E12.5. **(D)** Heatmap depicting outgoing and incoming signaling activities of major pathways across cell compartments at E12.5. **(E)** Dot plot showing the expression levels and inferred interaction probabilities of the top predicted ligand-receptor pairs across key cell types at E12.5.

To further characterize compartment-specific signaling, we applied CellChat to DE-like cells, Non-DE-like cells, and dental mesenchymal cells. Inferred interaction heatmaps revealed a robust communication network, with the dental mesenchyme as the predominant sender and DE-like cells as the primary receiver (**Figures 4A–C**). The strongest inter-compartment axis was mesenchyme to DE-like signaling, consistent with mesenchymal induction of the odontogenic epithelium. Pathway-level decomposition indicated that extracellular matrix (ECM) and basement membrane signaling-particularly COLLAGEN-, FN1-, and AGRN-associated interactions-together with midkine/pleiotrophin (MK/PTN) signaling were major contributors. Reciprocally, DE-like cells signaled back to the mesenchyme primarily via platelet-derived growth factor (PDGF) signaling (**Figures 4D, E**).

**Figure 5:**
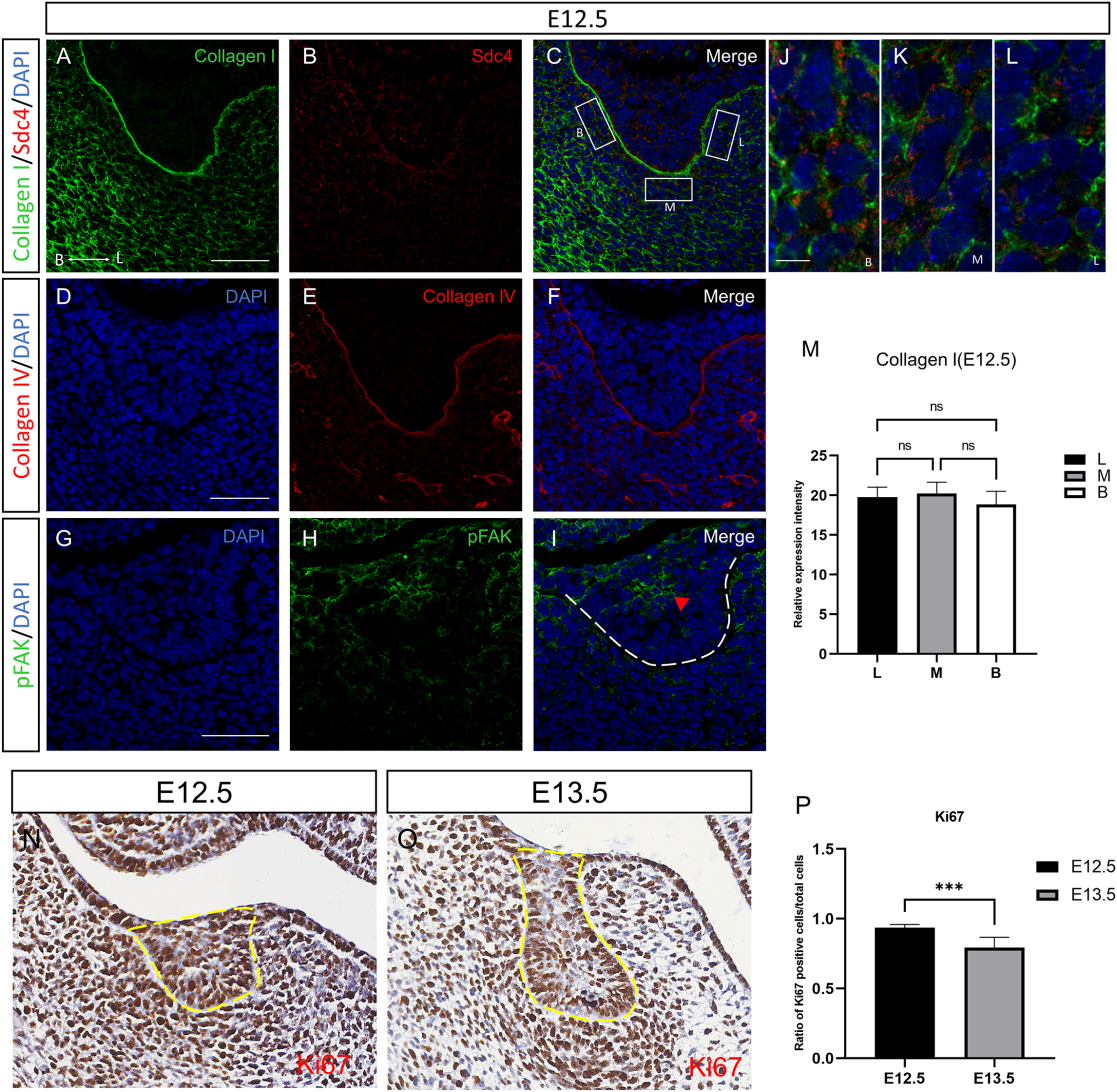
Spatial distribution of ECM- and adhesion-related markers and epithelial proliferation during early mandibular molar morphogenesis. **(A-I)** Immunofluorescence staining of Collagen I, Sdc4, pFAK, and Collagen IV in the dental lamina epithelium of the E12.5 mandibular first molar germ. **(J–L)** Higher-magnification views corresponding to boxes B, M, and L in panel C, respectively. Scale bars: 50μm **(A–I)** and 10μm **(J–L)**. **(M)** Quantification of relative fluorescence intensity in the mesenchyme adjacent to the buccal, lingual, and intermediate regions of the odontogenic epithelium at E12.5. Groups were compared using one-way ANOVA. ns, not significant. **(N, O)** Immunohistochemical staining for Ki67 in the odontogenic epithelium at E12.5 and E13.5. The yellow dashed line marks the invaginating dental lamina region. Scale bar= 50 μm. **(P)** Quantification of proliferating cells in the odontogenic epithelium at E12.5 and E13.5. Values represent mean ± SD. Data were analyzed using an unpaired Student’s t-test. ***P < 0.001.

To relate transcriptomic and inferred communication features to tissue-level organization at E12.5, we examined epithelial-mesenchymal interface markers by immunofluorescence and performed *in situ* hybridization for Epha4. Collagen I signal was enriched in the odontogenic mesenchyme adjacent to the dental lamina epithelium, with comparable patterns on the buccal and lingual sides (**Figures 5A–C, J-M**). Collagen IV localized to the epithelial basement membrane and formed a continuous linear pattern along the epithelial-mesenchymal boundary, consistent with preserved basement membrane integrity (**Figures 5D–F**). Sdc4 staining was predominantly epithelial, with weaker signal in the mesenchyme (**Figures 5A–C**). Consistent with spatially patterned adhesion signaling, pFAK staining was increased in suprabasal epithelial cells and in the cervical region adjacent to the epithelial constriction (**Figures 5G–I**). Consistent with this proliferative state, Ki67 immunohistochemistry revealed widespread Ki67-positive cells in the E12.5 dental placode epithelium (**Figures 5N–P**). Epha4 signal was largely confined to the odontogenic epithelial domain, with weaker signal in non-odontogenic regions (**Figures 2A–C**). Together, these data suggest that the odontogenic epithelium at E12.5 is highly proliferative and poised for morphogenetic remodeling, in a context of prominent mesenchymal ECM- and MK/PTN-associated signaling. This state is temporally associated with mesenchymal ECM- and MK/PTN-related signaling and, in CellChat inference, accompanied by PDGF-associated feedback from DE-like cells to the mesenchymal niche, consistent with the stage of dental lamina thickening and early epithelial invagination.

### ECM-adhesion signaling and focal adhesion-related programs are prominent in DE-like cells at E13.5

At E13.5, we identified 358 differentially expressed genes between DE-like and Non-DE-like cells; 320 genes were upregulated and 38 were downregulated in DE-like cells (**Figures 2G–I**). The upregulated gene set was enriched for markers of a mechanically reinforced epithelial program, including keratins (*Krt5, Krt17*) and basement membrane components (*Fras1, Frem2, Npnt*) (**Supplementary Table 2**). GO enrichment implicated Wnt signaling, epithelial proliferation, and cell-substrate adhesion, with overrepresented cellular components related to the extracellular matrix (ECM), basement membrane, and focal adhesions; enriched molecular functions included adhesion-related binding and growth factor receptor activity (**Figure 2J**). Consistently, KEGG pathway analysis identified enrichment of Wnt signaling, focal adhesion, PI3K–Akt, MAPK, and Rap1 pathways, as well as tight junction and Hedgehog signaling (**Figure 2K**), suggesting coordinated remodeling of epithelial adhesion and junctional regulation. *In situ* hybridization further showed that Epha4 transcripts were predominantly localized to the odontogenic epithelium at E13.5, with weaker signal in adjacent non-odontogenic regions (**Figures 2D–F**).

**Figure 6:**
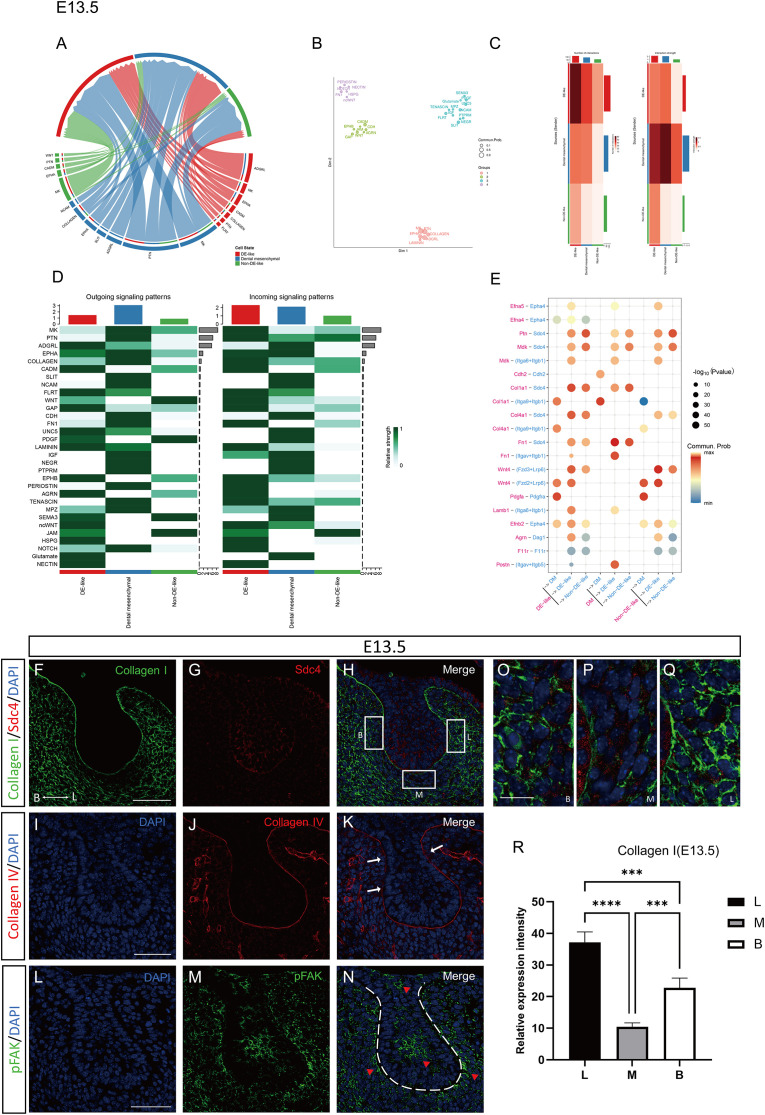
ECM-, Sdc4-, and pFAK-associated signaling features and spatial remodeling patterns at E13.5. **(A)** Chord diagram showing all signaling pathways with significant communication probabilities (p < 0.05) among DE-like cells, Non-DE-like cells, and dental mesenchyme. **(B)** Communication network of signaling pathways at E13.5, in which each node represents a signaling pathway and shorter distances indicate greater similarity in intercellular communication patterns. **(C)** Heatmap of inferred intercellular communication networks among major cell compartments at E13.5. **(D)** Heatmap depicting outgoing and incoming signaling activities of major pathways across cell compartments at E13.5. **(E)** Dot plot illustrating the expression patterns and interaction probabilities of the top predicted ligand-receptor pairs across key cell types at E13.5. **(F-N)** Immunofluorescence staining of Collagen I, Sdc4, pFAK and Collagen IV in the dental lamina epithelium of the mandibular first molar germ at E13.5. **(O-Q)** Higher-magnification views corresponding to boxed regions B, M, and L in panel **(H)**, respectively. Arrows in panel K indicate regions of basement membrane discontinuity, while red arrowheads in panel M denote areas with high pFAK expression. Scale bars: 50μm in panels **(F-N)** and 10μm in panels **(O-Q)**. **(R)** Statistical analysis of relative expression intensity in mesenchymal regions adjacent to the odontogenic epithelium (buccal, lingual, and intermediate regions) at E13.5. Groups were compared using one-way ANOVA. ***P < 0.001, ****P < 0.0001.

To interrogate tissue-level interactions, we inferred intercellular communication with CellChat. Among the highest-ranking ligand-receptor pairs, ECM ligands (collagens, laminins, fibronectin, and agrin) were preferentially enriched in mesenchyme-to-epithelium signaling, suggesting the involvement of an ECM-receptor-focal adhesion-associated signaling module (**Figures 6A–D**). These interactions included integrin pairs and the proteoglycan receptor Sdc4, consistent with focal adhesion-associated signaling during epithelial invagination. Eph/ephrin signaling, including Efna5-Epha4 and Efnb2-Epha4, was also enriched, suggesting potential involvement in boundary specification at the epithelial-mesenchymal interface. In addition, Wnt4-FZD3/LRP6, PDGFA-PDGFRA, and Postn-ITGAV/ITGB5 interactions highlighted coupling between epithelial programs and the ECM and underscored mesenchymal contributions during tooth morphogenesis (**Figure 6E**).

We then examined the spatial distribution of ECM/adhesion markers and related readouts by immunofluorescence and *in situ* hybridization to relate these transcriptomic signatures to tissue-level morphogenesis. Collagen I immunoreactivity increased in the odontogenic mesenchyme adjacent to the epithelium and was stronger on the lingual side, indicating the emergence of buccolingual ECM asymmetry (**Figures 6F–H, O-R**). Because the dissociated scRNA-seq datasets do not preserve explicit buccolingual spatial coordinates, the present analysis cannot determine whether this regional mesenchymal pattern is primarily induced by asymmetric epithelial cues, reflects an intrinsic mesenchymal prepattern, or arises through reciprocal reinforcement between both compartments. Collagen IV labeling showed focal discontinuities at the cervical constriction and along the buccal aspect of the basement membrane (**Figures 6I–K**). Sdc4 staining was detected in both basal and suprabasal layers, with higher intensity in basal cells (**Figures 6F–H**). Phosphorylated FAK (pFAK) exhibited a polarized distribution, with elevated signal on the lingual and apical sides of the tooth bud, spatially coinciding with the leading edge of the invaginating suprabasal epithelium (**Figures 6L–N**).

### E14.5 DE-like epithelium exhibits odontogenic differentiation and adhesion remodeling

At E14.5, a total of 1297 differentially expressed genes (DEGs; FDR-adjusted P < 0.05) were identified between DE-like and non-DE-like epithelial cells, comprising 546 upregulated and 751 downregulated genes (**Figures 3A–C**). The upregulated gene set was enriched for markers of a more differentiated odontogenic epithelial phenotype, including key developmental regulators and morphogenetic pathways. DE-like cells maintained epithelial identity markers (e.g., *Pitx2, Lef1, and Epcam*) and displayed transcriptional signatures consistent with increased FGF/MAPK responsiveness. Additionally, these cells upregulated adhesion and structural scaffold components (*Itga6 and Itgb5)*, consistent with enhanced cell-matrix interactions and a more stabilized tissue architecture during morphogenesis (**Supplementary Table 3**). GO enrichment analysis of the upregulated genes highlighted processes involved in Wnt signaling, morphogenesis of an epithelial fold and actin filament organization. Enrichment of cellular components emphasized the basement membrane, collagen-containing extracellular matrix, adherens junction, and focal adhesions, suggesting their role in remodeling the epithelial–mesenchymal interface during odontogenesis (**Figure 3D**). KEGG pathway analysis identified enrichment of focal adhesion and ECM-receptor interactions, focusing on pathways such as PI3K-Akt, Wnt, Rap1, and adherens signaling, suggesting coordinated remodeling of epithelial adhesion and junctional regulation (**Figure 3E**).

**Figure 7:**
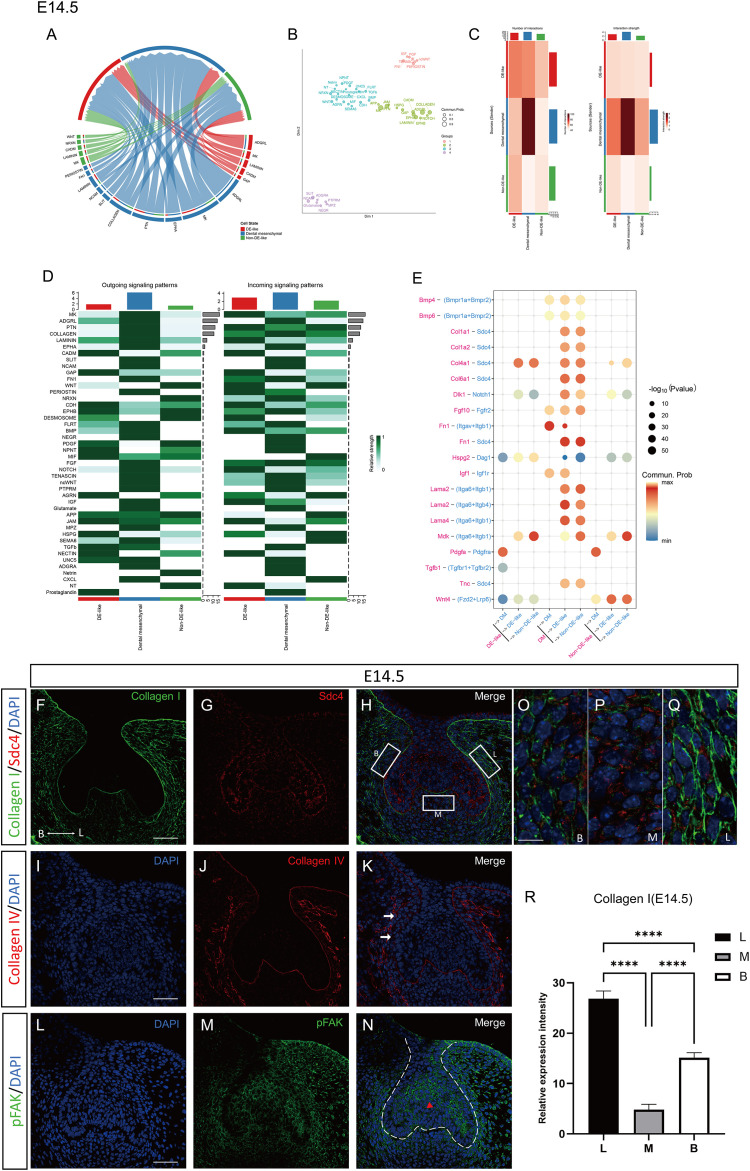
Cell-cell communication and ECM-adhesion-associated spatial features in DE-like cells at E14.5. **(A)** Chord diagram showing all signaling pathways with significant communication probabilities (p < 0.05) among DE-like epithelial cells, non-DE-like epithelial cells, and dental mesenchyme. **(B)** Communication network of signaling pathways at E14.5, in which each node represents a signaling pathway and shorter distances indicate greater similarity in intercellular communication patterns. **(C)** Heatmap of inferred intercellular communication networks among major cell compartments at E14.5. **(D)** Heatmap depicting outgoing and incoming signaling activities of major pathways across cell compartments at E14.5. **(E)** Dot plot illustrating the expression patterns and interaction probabilities of the top predicted ligand–receptor pairs across key cell types at E14.5. **(F-N)** Immunofluorescence staining of Collagen I, Sdc4, pFAK and Collagen IV in the dental lamina epithelium of the mandibular first molar germ at E14.5. **(O-Q)** Higher-magnification views corresponding to boxed regions B, M, and L in panel **(H)**, respectively. Arrows in panel K indicate regions of basement membrane discontinuity, while red arrowheads in panel M denote areas with high pFAK expression. Scale bars: 50μm in panels **(F-N)** and 10μm in panels **(O-Q)**. **(R)** Statistical analysis of relative expression intensity in mesenchymal regions adjacent to the odontogenic epithelium (buccal, lingual, and intermediate regions) at E14.5. Groups were compared using one-way ANOVA. ****P < 0.0001.

CellChat analysis positioned DE-like cells as major receivers of mesenchymal-derived signals, particularly MK/PTN signaling, with ECM components (e.g., Collagen, Laminin, Periostin, FN1) converging on adhesion receptors such as Sdc4 and integrin complexes (**Figures 7A–C**). These findings suggest that these pathways are associated with DE-like cell adhesion-related programs and communication within the mesenchymal niche during odontogenesis (**Figures 7D, E**). Eph/Ephrin signaling, particularly the Efna5-EPHA4 interaction, further supported the association of EPHA4 with a boundary-related epithelial-mesenchymal contact zone (**Figure 7E**).

At the protein level, Collagen I was predominantly expressed in the odontogenic mesenchyme adjacent to the epithelium, with stronger expression on the lingual side, indicating maintenance of regional mesenchymal ECM asymmetry during early cap morphogenesis (**Figures 7F–H, O-R**). However, the initiating source of this asymmetry cannot be resolved from the current dataset. Collagen IV expression was absent at the cervical loop and lingual side, suggesting remodeling at these sites (**Figures 7I–K**), in contrast to its continuous expression during earlier stages (E12.5-E13.5). Sdc4 expression was elevated in basal dental epithelial cells and stellate reticulum precursors, consistent with a role in epithelial-mesenchymal interactions during odontogenesis (**Figures 7F–H**). Additionally, pFAK expression was high in stellate reticulum precursors, consistent with ongoing morphogenetic remodeling in these regions (**Figures 7L–N**). Notably, pFAK expression was absent in the enamel knot, a structure involved in signaling during tooth cusp formation, highlighting its distinct expression pattern compared to earlier stages.

**Figure 8:**
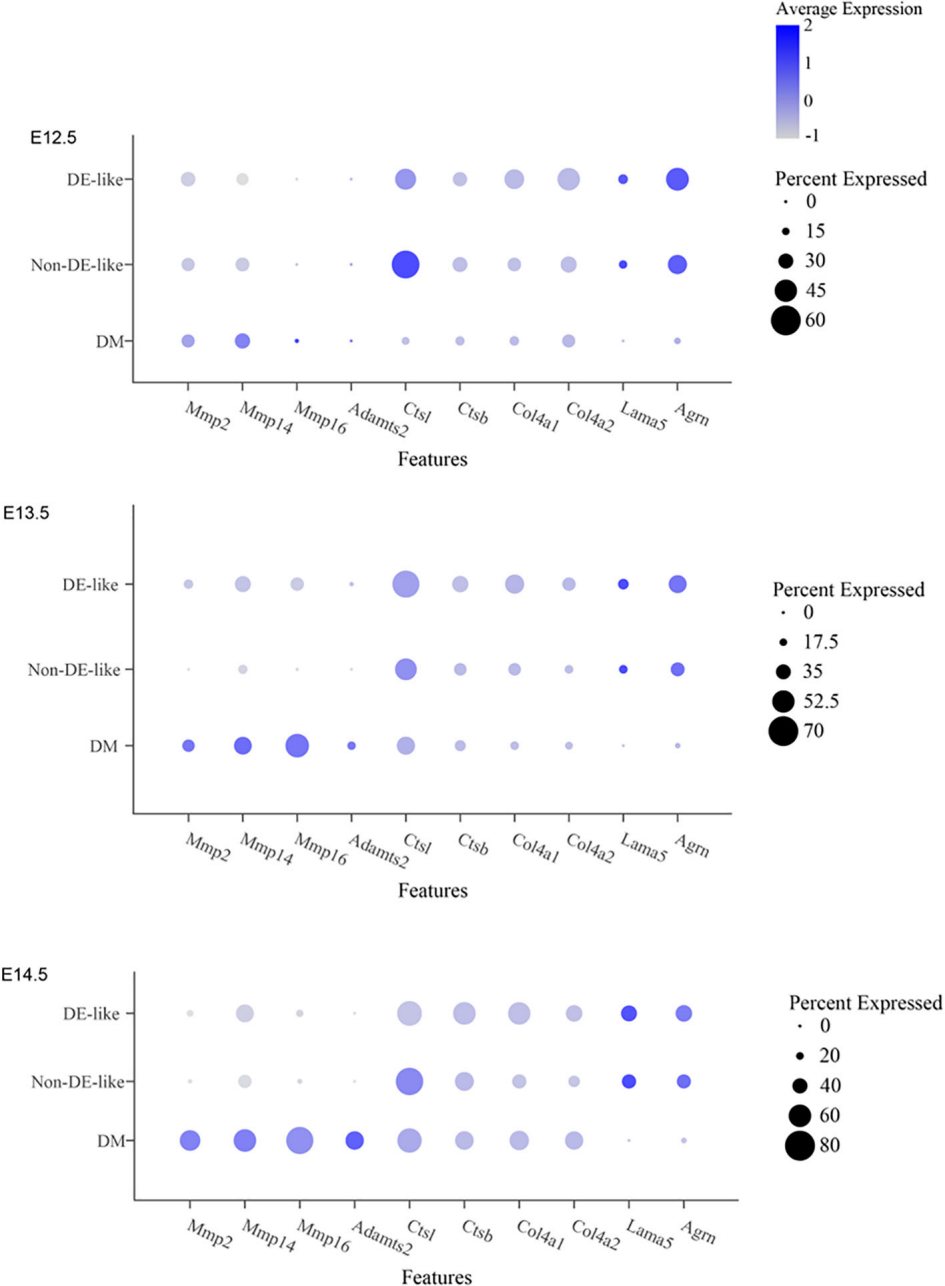
Stage-resolved expression of candidate ECM-remodeling proteases and basement membrane-associated genes across E12.5-E14.5.

To further investigate candidate drivers of the focal collagen IV discontinuities, we examined the scRNA-seq datasets for ECM-remodeling proteases and basement membrane-associated genes across E12.5-E14.5. Rather than identifying a single dominant protease in DE-like cells, this analysis revealed a stage-resolved remodeling-permissive microenvironment at the epithelial-mesenchymal interface. Mesenchymal populations showed detectable expression of Mmp2, Mmp14, Mmp16, and Adamts2, whereas Ctsl was broadly expressed across epithelial and mesenchymal compartments. In parallel, transcripts encoding basement membrane components, including Col4a1, Col4a2, Lama5, and Agrn, remained detectable in epithelial cells. Together, these findings support localized matrix turnover at the epithelial-mesenchymal interface, although the precise spatial enrichment of individual proteases at the cervical loop remains to be determined (**Figure 8**).

Taken together, these stage-resolved transcriptomic, morphometric, and spatial observations identify a candidate ECM-Sdc4-FAK-associated module that is temporally and spatially linked to epithelial invagination and the emergence of buccolingual asymmetry during early mandibular molar morphogenesis. However, because the present study does not include direct perturbation of FAK or Sdc4, these data support an association model rather than establishing pathway necessity.

## Discussion

Tooth morphogenesis between E12.5 and E14.5 represents a critical window for understanding the dynamics of epithelial shape changes, while preserving the integrity of the epithelial-mesenchymal interface and initiating spatially restricted signaling. By integrating morphometric measurements, reanalyzed single-cell transcriptomes, inferred ligand-receptor communication, and spatial validation of ECM and adhesion markers, we delineate a basement membrane-Sdc4-pFAK signaling framework associated with mandibular first molar invagination. Our data suggest that Sdc4-associated matrix engagement and pFAK-linked adhesion signaling coincide with (1) placode thickening during bud invagination, (2) basement membrane remodeling at the cervical constriction, and (3) the emergence of bucco-lingual asymmetry as the cap stage approaches.

A key strength of this study is the biological reinterpretation of public stage-resolved scRNA-seq resources within a unified analytical framework. Instead of generating another descriptive atlas, we focused specifically on epithelial state transitions across the E12.5-E14.5 interval. Combining epithelial subclustering, stage-specific differential expression, cell-cell communication analysis, morphometric assessment, and spatial validation allowed us to characterize a temporally ordered shift from proliferation-dominant states to ECM-adhesion remodeling and then to a more stabilized tissue state. This stage-dependent progression provides a useful framework for understanding how epithelial shape changes are coordinated during early molar development.

Building on current models of tooth morphogenesis, we find that invagination cannot be solely attributed to proliferation. Instead, it involves coordinated cellular rearrangements, including suprabasal intercalation, migration, and vertical telescoping (Li et al., 2020; Wang et al., 2022a; Novacescu et al., 2025). Inhibition of focal adhesion kinase (FAK) further emphasizes the role of focal adhesion signaling in the bud-to-cap transition, underscoring its functional involvement in invagination (Yamada et al., 2019). Building on these observations, our analysis points to a basement membrane-Sdc4-pFAK interface that may help organize epithelial remodeling during invagination (Vignes et al., 2022). This module may provide a framework linking patterned signaling with tissue mechanics through an ECM-constrained interface, potentially contributing to the spatiotemporal coordination of epithelial morphogenesis (Katoh, 2026). This framework may help explain how traction, migration, and epithelial stratification are localized while epithelial integrity is preserved (Höfs and Wickström, 2024). In contrast to growth-centric models, this receptor-mediated mechanotransduction framework may help explain how ECM cues could be translated into localized epithelial behaviors, including traction, migration, and stratification, while preserving epithelial integrity (Katoh, 2026). This framework also helps explain how the epithelium may respond to regional differences in ECM composition, including collagens, laminins, fibronectin, and agrin, and switch between states of stable anchorage and localized remodeling as invagination proceeds (Dibus et al., 2024).

Our observations from E12.5 to E13.5 revealed significant changes in invagination depth and cervical narrowing, reflecting rapid epithelial deformation. At E12.5, collagen IV remains intact along the basement membrane, with enriched Sdc4 and enhanced pFAK expression in suprabasal cells at the cervical region, where force transmission is crucial. By E13.5 and E14.5, transcriptomic data revealed enrichment of adhesion-related pathways, suggesting that later morphogenesis may increasingly rely on adhesion-regulated signaling in addition to proliferation-related programs (Di-Luoffo et al., 2021). This shift marks the transition from growth-driven to adhesion-driven morphogenesis, where initial epithelial growth supports placode thickening, while subsequent shape changes depend on a mechanochemical interface that integrates ECM signals and cytoskeletal remodeling (Vignes et al., 2022).

Supporting this model, our CellChat analyses consistently identified the dental mesenchyme as the dominant signal sender and DE-like cells as the primary receivers, with ECM- and basement membrane-associated pathways (e.g., collagens, laminins, FN1, agrin) and MK/PTN signaling prominently featured. Notably, the inferred ligand-receptor pairs at E13.5 include integrin complexes (e.g., *Itga9-Itgb1, Itga6-Itgb1*) and proteoglycan-associated receptors such as Sdc4, supporting a model in which Sdc4 acts as a co-receptor, concentrating ECM and heparin-binding cues at the basal surface to facilitate focal adhesion signaling (Chastney et al., 2021). At E12.5, DE-like cells exhibit PDGF signaling directed toward the mesenchyme, suggesting a possible feedback loop in which epithelial responses to mesenchymal ECM cues may influence the mesenchymal niche-potentially influencing ECM deposition, condensation, or differentiation (Xu et al., 2005). These computationally inferred communications are supported by spatially resolved changes in ECM deposition (Col1a1 enrichment in adjacent mesenchyme), basement membrane integrity (collagen IV continuity at E12.5, discontinuities at E13.5, and focal loss by E14.5), and adhesion signaling (polarized pFAK expression at the leading invagination front). Notably, the focal loss of collagen IV at the cervical loop was not accompanied by complete disappearance of basement membrane-associated transcripts in epithelial cells. Instead, the data are more consistent with localized matrix turnover or altered matrix assembly than with global shutdown of basement membrane production. Our survey of ECM-remodeling proteases supports this interpretation by identifying a remodeling-permissive microenvironment at the epithelial-mesenchymal interface. Collectively, these findings support the notion that pFAK marks the functional engagement of the epithelium with the surrounding matrix, while Sdc4 modulates both ECM sensing and signal amplification (Chronopoulos et al., 2020; Katoh, 2025).

Our data reveal a stage- and compartment-specific distribution of phosphorylated FAK (pFAK). pFAK was prominent in invagination-associated epithelial regions and stellate reticulum precursors but was not detected in the enamel knot at E14.5. These observations suggest that FAK-dependent mechanotransduction may be selectively engaged in epithelial regions requiring cell-matrix coupling and attenuated in contexts where alternative signaling pathways or cell-cell interactions predominate (Seetharaman and Etienne-Manneville, 2018). Enamel knot cells may reduce basement-membrane engagement and rely more heavily on cell–cell interactions to pattern the cusp (Jussila and Thesleff, 2012). In contrast, stellate reticulum precursors undergo substantial tissue rearrangement during early cap formation and may therefore rely more on matrix-associated mechanics (Balic and Thesleff, 2015). Accordingly, pFAK can serve as a readout of adhesion-mediated mechanical activity, being elevated in regions where matrix traction is required (Duda et al., 2019). Similarly, Sdc4 was enriched in basal epithelial layers, consistent with roles in ECM binding, co-receptor activity for heparin-binding ligands, and stabilization of adhesion signaling platforms. Elevated Sdc4 in the odontogenic epithelium suggests an increased requirement to integrate niche-derived cues (e.g., ECM and MK/PTN signals) with adhesive-state transitions (Chronopoulos et al., 2020).

Morphometric analyses showed that early morphogenesis (E12.5-E13.5) was characterized by pronounced invagination and lingual cervical narrowing, followed by refinement of buccolingual asymmetry at later stages (E13.5-E14.5). These trends coincided with regional differences in ECM distribution and basement membrane continuity, including stronger mesenchymal collagen I signal on the lingual side and focal collagen IV discontinuities at E13.5, particularly near cervical and buccal regions. Such regional ECM differences may impose anisotropic mechanical constraints that the epithelium must interpret (Kim et al., 2025). As ECM composition changes in the lingual mesenchyme, Sdc4 engagement and pFAK activation may be modulated. Notably, pFAK displayed a polarized distribution at E13.5 toward the lingual and apical aspects of the tooth bud, consistent with a mechanically reinforced interface that may support invagination and asymmetry. Together, our data suggest that regional ECM-state differences can pattern adhesion-related signaling in the odontogenic epithelium, thereby influencing directional morphogenesis and compartmentalization. An unresolved question raised by our data is what initiates the lingual-biased mesenchymal collagen I pattern observed at E13.5-E14.5. At present, our analyses do not allow us to distinguish whether this asymmetry is primarily induced by regional epithelial cues or reflects an intrinsic mesenchymal prepattern. These explanations are not mutually exclusive. One possibility is that asymmetric epithelial signaling progressively biases local mesenchymal ECM deposition and remodeling, thereby reinforcing regional matrix architecture or mechanical properties (Balic and Thesleff,2015; Du et al., 2024). An alternative model is that the dental mesenchyme is regionally prepatterned, either molecularly or mechanically, and that this anisotropic ECM state is subsequently interpreted and amplified by epithelial Sdc4-FAK-associated adhesion signaling during invagination (Chronopoulos et al., 2020; Vignes et al., 2022; Kim et al., 2025). Because the public scRNA-seq datasets used here were generated from dissociated cells and do not retain explicit buccolingual coordinates, and because the present study did not include compartment-specific perturbation, the causal hierarchy between epithelial signaling and mesenchymal ECM asymmetry remains unresolved. We therefore interpret the lingual-enriched Collagen I pattern as a spatial correlate of morphogenesis and a hypothesis-generating feature for future functional studies. Eph/ephrin signaling, particularly the Efna5-Epha4 interaction, is implicated in boundary specification and may intersect with the Sdc4-pFAK module to regulate adhesion at the epithelial–mesenchymal interface (Bush, 2022). This interaction could refine functional territories during the bud-to-cap transition. Future work will test whether Eph-mediated cues modulate integrin/Sdc4-driven adhesion signaling.

A limitation of the present study is that it is based on integrative reanalysis of public scRNA-seq datasets, inferred ligand-receptor communication, morphometric analysis, and spatial validation, but does not include direct perturbation of Sdc4 or FAK signaling. Therefore, the current data do not establish that the ECM-Sdc4-FAK module is necessary for epithelial invagination or buccolingual asymmetry. Instead, they support a candidate mechanochemical framework that is temporally and spatially linked to these morphogenetic events. Future studies using tooth germ explant culture, pharmacological FAK inhibition, and Sdc4 loss-of-function approaches will be required to directly test causality and determine whether this pathway is required for bud-to-cap transition and regional morphogenesis.

In conclusion, our study supports a candidate mechanochemical framework involving Sdc4, focal adhesion kinase (FAK), and basement membrane remodeling that is linked to epithelial invagination during early tooth morphogenesis. By coupling ECM-derived cues to cellular behaviors (e.g., migration and stratification), this module may contribute to tissue architecture remodeling and the emergence of buccolingual asymmetry during mandibular first molar development. Nevertheless, the precise regulatory logic governing cell-cell and cell-matrix interactions-including signaling exchanges among cell types and the temporal dynamics of ECM remodeling-remains incompletely defined and warrants further investigation.

## Data Availability

The original contributions presented in the study are included in the article/**Supplementary Material**. Further inquiries can be directed to the corresponding author.
